# Skin Lesion Classification Through Test Time Augmentation and Explainable Artificial Intelligence

**DOI:** 10.3390/jimaging11010015

**Published:** 2025-01-09

**Authors:** Loris Cino, Cosimo Distante, Alessandro Martella, Pier Luigi Mazzeo

**Affiliations:** 1 Dipartimento di Ingegneria Informatica, Automatica, e Gestionale “Antonio Ruberti”, Sapienza Università di Roma, Via Ariosto, 25, 00185 Roma, Italy; 2 Istituto di Scienze Applicate e Sistemi Intelligenti (ISASI), Consiglio Nazionale delle Ricerche (CNR), DHITECH, Campus Università del Salento, Via Monteroni s.n., 73100 Lecce, Italy; cosimo.distante@cnr.it (C.D.); pierluigi.mazzeo@cnr.it (P.L.M.); 3 Dermatologia Myskin, Poliambulatorio Specialistico Medico-Chirurgico, 73030 Tiggiano, Italy; alessandro.martella@dermatologiamyskin.it

**Keywords:** skin disease classification, skin dataset, test time augmentation, explainable artificial intelligence, explanatory task, convolution neural network

## Abstract

Despite significant advancements in the automatic classification of skin lesions using artificial intelligence (AI) algorithms, skepticism among physicians persists. This reluctance is primarily due to the lack of transparency and explainability inherent in these models, which hinders their widespread acceptance in clinical settings. The primary objective of this study is to develop a highly accurate AI-based algorithm for skin lesion classification that also provides visual explanations to foster trust and confidence in these novel diagnostic tools. By improving transparency, the study seeks to contribute to earlier and more reliable diagnoses. Additionally, the research investigates the impact of Test Time Augmentation (TTA) on the performance of six Convolutional Neural Network (CNN) architectures, which include models from the EfficientNet, ResNet (Residual Network), and ResNeXt (an enhanced variant of ResNet) families. To improve the interpretability of the models’ decision-making processes, techniques such as t-distributed Stochastic Neighbor Embedding (t-SNE) and Gradient-weighted Class Activation Mapping (Grad-CAM) are employed. t-SNE is utilized to visualize the high-dimensional latent features of the CNNs in a two-dimensional space, providing insights into how the models group different skin lesion classes. Grad-CAM is used to generate heatmaps that highlight the regions of input images that influence the model’s predictions. Our findings reveal that Test Time Augmentation enhances the balanced multi-class accuracy of CNN models by up to 0.3%, achieving a balanced accuracy rate of 97.58% on the International Skin Imaging Collaboration (ISIC 2019) dataset. This performance is comparable to, or marginally better than, more complex approaches such as Vision Transformers (ViTs), demonstrating the efficacy of our methodology.

## 1. Introduction

Non-melanoma and melanoma skin cancers account for over 1.5 million new cases (excluding basal cell carcinoma) and 120,000 deaths annually, with incidence rates approximately twice as high among men as among women [1]. Many scientific studies [2,3] have demonstrated that the only way to reduce the mortality rate from skin cancer is through early diagnosis. One of the most common methodologies to diagnosis skin cancer is dermoscopy; this technique captures skin images without surface reflections [4], and over the years, it has been widely employed in the diagnosis of skin lesions [5]. In clinical practice, dermatologists typically evaluate these dermoscopic images via visual inspection, which is time-consuming and highly dependent on each physician’s skill level. Even well-trained experts find it challenging to reliably distinguish between different skin conditions. Consequently, the development of automated methods for early and accurate skin lesion classification is of paramount importance.

In recent years, many computer-aided diagnosis (CAD) systems based on dermoscopic images have been proposed [6,7,8,9,10,11]. Most of these methods are powered by Convolutional Neural Networks (CNNs), where large, labeled datasets of skin lesions are used to train deep models. CNN-based techniques have significantly improved classification performance in CAD systems [12].

Data augmentation is frequently employed to enhance model accuracy by addressing the problem of limited training data, which is common in skin lesion analysis but less so in other domains such as ImageNet [13]. Top-ranked ISIC Challenge submissions routinely adopt data augmentation [14,15,16], and specific augmentation strategies have been extensively investigated in the literature [17,18,19]. For instance, Vasconcelos and Vasconcelos [18] report improvements using geometric transformations (rotations, flips, lesion-preserving crops), PCA-based color augmentation, and specialized warping that preserves lesion symmetries. Similarly, Pham et al. [19] compare different forms of data augmentation for classifiers (SVMs, neural networks, and random forests) trained on features extracted using a pre-trained Inception-v4 network. Srinivasu et al. [20] further show how different augmentation strategies can balance various lesion classes in the training set.

Despite the success of CNN-based CAD systems, interpretability remains a significant concern, particularly for clinical use [21,22]. During training, CNNs learn high-level feature representations to optimize classification accuracy, but these internal processes are largely opaque. Dermatologists and other medical practitioners must often rely solely on a model’s output probabilities without a clear understanding of how specific image regions drive these decisions. Techniques such as Gradient-weighted Class Activation Mapping (Grad-CAM) can help mitigate this problem by providing a visual explanation of the features guiding the network’s predictions.

Regulatory requirements worldwide, including those proposed by the EU commission, increasingly demand transparency in medical software, including CAD systems used as medical devices [23]. To meet these expectations, explainable artificial intelligence (xAI) methods are being explored as a means of clarifying how deep learning architectures arrive at diagnostic decisions [24,25,26,27]. The importance and challenges of xAI in the medical domain are widely debated, with ongoing efforts to establish conceptual frameworks for explainable AI in healthcare [28,29,30].

Several motivations drive this study. First, we aim to compare multiple CNN backbones to determine their effectiveness in identifying and classifying different skin lesions. Second, we investigate how Test Time Augmentation (TTA) influences classification performance when integrated into the post-processing stage of trained networks. Finally, we incorporate xAI-based techniques to offer visual justifications for the networks’ classification outcomes, aiding clinicians in understanding both successful and erroneous diagnoses.

In light of these objectives, this work offers the following contributions:

- Comparison of CNN Backbones: We investigate six well-known architectures— ResNeXt50 [31], ResNet152 [32], and EfficientNetB4–B7 [33]—each appended with a fully connected layer, for the automatic classification of skin lesions. We evaluate their performance in terms of balanced multi-class accuracy (BCA) on the publicly available ISIC 2019 dataset.
- Test Time Augmentation (TTA): We demonstrate that the use of TTA in the post-processing phase significantly improves classification accuracy by reducing misclassifications, comparing the performance of each architecture with and without TTA.
- Explainable AI (xAI) Techniques: We explore the main regions of skin lesions involved in the classification process using t-SNE and Grad-CAM. By visually explaining why misclassifications occur, we encourage the deployment of these trained architectures—accompanied by transparent justifications—in clinical settings.
- Performance on ISIC 2019: Based on empirical results, our architectures achieve performance levels on par with more complex models, with a BCA of 97.58% on the ISIC 2019 dataset.

Overall, this work aims to improve both the performance and interpretability of automated skin lesion classification systems, thereby enhancing their reliability and acceptance in clinical workflows.

### 1.1. Related Work

This section reviews related works from the literature, organized according to their primary contributions. For a more in-depth study of deep learning techniques, Adegun and Viriri [34] provide a survey of state-of-the-art deep learning techniques for skin lesion analysis and melanoma detection, offering insights into various methodologies and their performance.

**Early Deep Learning-Based Approaches:** Many techniques for automatic skin lesion classification have been developed and applied in the healthcare field over the past decade. Early studies primarily focused on leveraging deep CNNs to classify skin diseases. For instance, Chowdhury et al. [35] utilized a custom CNN trained on the HAM10000 dataset [26] to detect seven types of skin disorders. Similarly, Esteva et al. [36] employed CNNs on the ISIC 2018 dataset and other private data with backpropagation [37] as an interpretability tool, achieving effective multi-class classification results. Li et al. [38] combined VGG16 [39] and ResNet-50 [32] models in an ensemble fashion on the ISIC 2018 dataset, using occlusion analysis [40] for explanation.

**Integration of Data Pre-processing and Segmentation:** Several methods improved performance through data pre-processing and lesion segmentation techniques. Kassani and Kassani [41] explored multiple deep learning architectures for melanoma detection, employing pre-processing methods to reduce noise and data augmentations. Salido and Ruiz [42] proposed a method that automatically segments lesions and removes occlusions (e.g., hair) before applying a deep CNN. Similarly, Shahin et al. [43] introduced data augmentation to reduce overfitting and improve classification accuracy, and Sherif et al. [44] utilized a deep CNN for melanoma detection. Object detection and segmentation methods have also been integrated, as in Ünver and Ayan [45], which employed YOLO and GrabCut to isolate melanoma-affected regions for further classification.

**Advanced Architectures and Enhanced Feature Extraction:** Recent works introduced advanced architectures to improve intra-class consistency and inter-class discrimination. For example, Wang et al. [46] employed a CAM-based approach to refine class activation maps, enhancing classification accuracy. Qian et al. [47] integrated multi-scale attention blocks and class-specific loss weighting, while Alenezi et al. [48] combined wavelet transforms, pooling, and normalization layers within a residual neural network. Other methods have aimed at improving segmentation and classification synergy. Anand et al. [49] merged U-Net and CNN models to enhance both lesion segmentation and classification, and Nakai et al. [50] introduced the Enhanced Deep Bottleneck Transformer (EnDBoT) with a novel Dual Position encoding Self-Attention block to refine feature learning.

**Utilization of Test Time Augmentation (TTA) and Data Augmentation:** Data augmentation and TTA techniques have been shown to improve generalization and robustness. Ashraf et al. [51] applied TTA and a conditional random field (CRF) in a post-processing step to boost segmentation accuracy. Similarly, jiahao et al. [52] combined EfficientNet models with TTA for improved skin cancer classification. Lee [53] explored uncertainty estimation using test time mixup augmentation, while Perez et al. [54] studied the effect of different data augmentation strategies on melanoma classification. Valle et al. [17] emphasized the importance of data augmentation, demonstrating that predictions averaged over 50 augmented test samples yield more reliable results. Such augmentation strategies are crucial in mitigating overfitting and improving model robustness.

**Explainable AI (xAI) Techniques:** Explainability is critical for clinical adoption. Various studies integrated xAI methods such as CAM [25], Grad-CAM [24], and occlusion analysis [40] to provide interpretability. Singh et al. [55] proposed a two-stage pipeline with Test Time Augmentation and saliency-based explainers, including XRAI, Grad-CAM, and Guided Backprop, to clarify model decisions. Zhou et al. [25] introduced CAM for object localization, later adopted by Li et al. [56], Xie et al. [10], Yang et al. [57], and Zunair and Hamza [58] to identify and classify skin diseases. Additional explainability methods, such as Content-Based Image Retrieval (CBIR) [59], Kernel SHAP [60], and fuzzy decision trees [61], have also been incorporated. These xAI tools help clinicians understand why a model makes a particular prediction, increasing trust and facilitating medical decision-making.

**Emerging Trends and Comparative Analyses:** Several contemporary works focus on combining multiple approaches or introducing new architectures. Zeng et al. [62] presented a distillation learning approach for skin disease classification, while Veeramani et al. [63] utilized a two-layer classifier incorporating the “F” flag feature. Some studies explore integrating additional data sources to enhance predictive performance [64,65]. Rezaee and Zadeh [66] proposed a multi-part model with transformers, convolutional networks, and self-attention units, and Ahmad et al. [67] introduced a Vision Transformer (ViT) [68] for both segmentation and classification tasks. Hybrid methods combining deep CNNs with machine learning classifiers, such as VGG-19 with SVM [69], further push the performance envelope.

**Performance Compared to Physicians:** Given the high stakes of dermatological diagnoses, several studies compare deep learning models to human experts, even early works as Esteva et al. [36]. A comprehensive survey by Haggenmüller et al. [70], analyzing 19 papers, found that in most cases, CNNs outperform physicians in diagnostic accuracy. This indicates the substantial clinical potential of these models. However, while accuracy is crucial, the ability to explain predictions and provide interpretable reasoning remains a significant challenge.

**Identified Gap and Our Contribution:** Despite the proliferation of advanced techniques, no existing solution simultaneously achieves high accuracy, strong interpretability, and the capability to offer meaningful insights into processed images. We combined most of the methodologies proposed in literature to deliver competitive performance aligned with state-of-the-art methods, while maintaining a high degree of interpretability. By focusing on an explainable design, we seek to facilitate better understanding and trust in model predictions, ultimately bridging the gap between black-box deep learning solutions and clinically acceptable, transparent decision-making.

## 2. Materials and Methods

In this section, we report our proposed method to use a deep architecture, a CNN-based backbone and a fully connected layer, for the categorization of skin diseases. Every experiment is conducted using the publicly accessible ISIC-2019 dataset. Section 2.1 contains a description of our model, and Section 2.2 contains a detailed breakdown of our fine-tuning approach. Section 2.3 contains a description of the Test Time Augmentation approach.

### 2.1. Model

**Figure 1:**
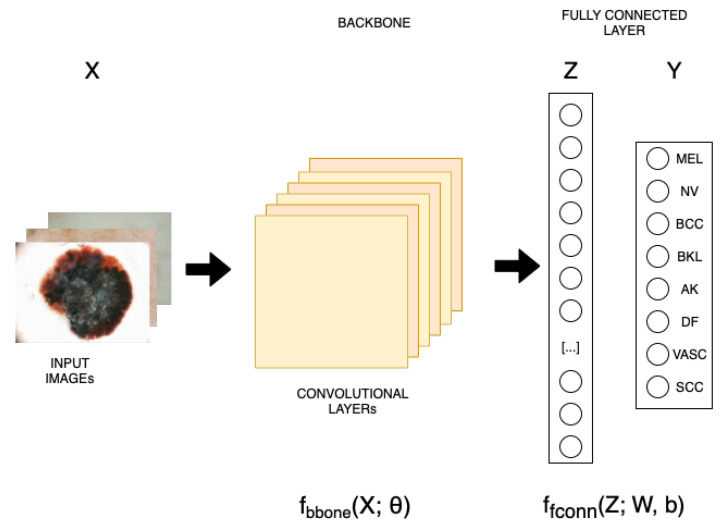
Proposed architecture. Beginning with a pre-trained architecture on the ImageNet dataset, we remove the last fully connected layer to configure the network to produce 8 output neurons. Subsequently, the entire network is re-trained using the ISIC 2019 dataset.

As described in Figure 1, the proposed solution is based on two main parts: a CNN backbone and a fully connected layer for classification. The CNN-based backbone block extracts the image features for classifying input according to the class they belong to. This problem is known as supervised classification, one of most common paradigms used for automatic object classification tasks. Given an image Given an image $x_{m}\in R^{W\times H\times 3}$, we extract the features $z_{m}\in R^{w\times h\times c}$, where w, h, c represent the output tensor dimensions of the last layer of the CNN backbone.

Considering $X={\left\{x_{m}\right\}}_{m=1}^{M}$ as the set of images and $Y={\left\{y_{m}\right\}}_{m=1}^{M}$ the respective labels, we want to find a function $f_{\mathrm{class}}$ that maps the image set X to its respective class labels Y. The function $f_{\mathrm{class}}$ is composed of two functions: the CNN backbone function $f_{\mathrm{bbone}}$ (see Equation (1)) and the fully connected layer function $f_{\mathrm{fconn}}$ (see Equation (2)).

(1) $$f_{bbone}:\;R^{W\times H\times 3}\to R^{w\times h\times c}\;\;|\;\;Z=f_{bbone}\left(X;\theta \right)$$

(2) $$f_{fconn}:\;R^{w\times h\times c}\to R^{C}\;\;|\;\;Y=f_{fconn}\left(Z;W,b\right)$$

where θ is the matrix of parameters of the CNN backbone and W and b are the hyperplane representation of the fully connected function. Combining these two equations, the backbone block and the fully connected layer, we want to find an overall function that, when given an image $x_{m}\in R^{W\times H\times 3}$ as input, maps it to a vector of class probabilities $p_{m}\in R^{C}$, where C is the number of classes:

(3) $$f_{\mathrm{class}}:\;R^{W\times H\times 3}\to R^{C}\;\;|\;\;f_{\mathrm{class}}\left(X;\theta ,W,b\right)=f_{\mathrm{fconn}}\left(f_{\mathrm{bbone}}\left(X;\theta \right);W,b\right)$$

We have investigated different well-known backbones in the scientific community such as ResNext, ResNet, and EfficientNet:

1. The ResNet152 [32] architecture family is one of the most common architectures for its flexibility to solve many tasks. The main characteristics introduced by DCNN are shortcut connections and residual learning. Shortcut connections help in solving the gradient vanishing problem for DCNN: some connections skip one or more layers. Residual learning also solves a problem with DCNN: experimental results show that going deeper with layers improves the performance but when the network is too deep, it loses generalization. Residual learning proposes learning the difference between the current state and the next rather than a new state for each layer to make the learning process more stable.
2. ResNeXt [31] introduced the concept of cardinality. The convolution filters are grouped in sets. The input of the following layer is only the output of the filter that belongs to the same group. This technique reduces the number of parameters required.
3. EfficientNet [33] The main characteristic of the EfficientNet family is that they have a compound coefficient that keeps the ratio of depth/width/resolution constant. EfficientNet is not much different from ResNet if we do not take into account this compound coefficient.

### 2.2. Fine-Tuning Approach

For computer vision problems, pre-training the model on a sizeable "upstream" dataset and fine-tuning it on a smaller target dataset is the most-used method for training deep architectures. Given a pre-trained CNN on a source dataset $D_{s}={\left\{\left(x_{s}^{i},y_{s}^{i}\right)\right\}}_{i=1}^{M}$, transfer learning aims to fine-tune to a target dataset $D_{t}={\left\{\left(x_{t}^{i},y_{t}^{i}\right)\right\}}_{i=1}^{N}$; generally, $D_{s}$ and $D_{t}$ share the same input image space X but have different class spaces $Y_{s}$ and $Y_{t}$. In general, $D_{s}$, being a large-scale dataset, lacks portability; only $D_{t}$ and a CNN pre-trained on $D_{t}$ are available during the fine-tuning task. In most computer vision applications, traditionally, $D_{s}$ denotes ImageNet [13] and $D_{t}$ is the smaller image target dataset linked to the visual classification problem we are investigating.

Since $Y_{s}$ and $Y_{t}$ are heterogeneous, $f_{\mathrm{class}}$ pre-trained on $D_{s}$ is not directly applicable to target data. As already explained in Section 2.1, we split $f_{\mathrm{class}}$ into two parts: a CNN-based backbone function $f_{\mathrm{bbone}}\left(X,\theta^{0}\right)$ (parameterized by $\theta^{0}$) and a task-specific function $f_{\mathrm{fconn}}^{s}\left(Z;W_{s}^{0},b_{s}^{0}\right)$ (parameterized by $W_{s}^{0}$ and $b_{s}^{0}$ in the source dataset) which denotes the last fully connected layer of the pre-trained model. We retain the CNN-based backbone function and replace the task-specific function with a randomly initialized function $f_{\mathrm{fconn}}^{t}\left(Z;W_{t},b_{t}\right)$ whose output space matches $Y_{t}$, where the $W_{t}$ and $b_{t}$ are the weights and biases of this fully connected layer, respectively. This gives rise to a fine-tuning strategy by solving Equation (5), where ℓ(·,·) is a loss function and weighted-cross entropy (WCE) is our choice for classification. This loss is a version of the cross-entropy that penalizes the error of some classes more than others. Usually, the weights are used to penalize more errors in the less frequent classes to balance the dataset.

The weights are proportional to the inverse of the number of images in the dataset for each class, as detailed in Equation (4)

(4) $$w_{j}=\frac{\sum_{i=1}^{C}c_{i}}{c_{j}}$$

where $w_{j}$ is the weight for the *j*-th class, $c_{j}$ is the number of images that belong to the *j*-th class, and C is the number of classes. The equation ensures that the weights add up to one so as not to change the value of the cost function. Remember that initialization matters in optimizing the model architecture because it is a non-convex optimization problem. Using pre-trained parameters $\theta_{0}$ is a good starting point for the optimization task.

(5) $$\left(\theta^{★},\theta_{t}^{★}\right)=\underset{\theta^{0},\theta_{t}}{\arg\min}\frac{1}{|D_{t}|}\sum_{i=1}^{N}\ell \left(f_{fconn}^{t}\left(f_{bbone}\left(x_{t}^{i},\theta^{0}\right);W_{t},b_{t}\right),y_{t}^{i}\right)$$

Here, $\theta^{★}$ represents the parameters of the backbone, and $\theta_{t}^{★}$ refers to the parameters of the last fully connected layer that minimizes the loss function. Besides this fine-tuning strategy, there are also techniques focusing on preventing overfitting.

**Figure 2:**
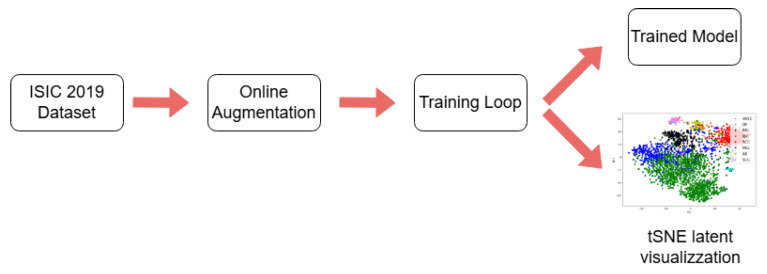
Training procedure. This methodology is applied to each model within the study. The outputs of this process include a trained model and a latent space visualization of the test set.

Figure 2 provides a concise overview of the proposed methodology. During the training phase, online augmentations are applied to the ISIC 2019 dataset to fine-tune several models based on different CNN backbones. Online augmentation is a data pre-processing technique applied dynamically during model training, where transformations or augmentations are performed on input images in real time. This approach allows for increased dataset diversity and improved model generalization without requiring prior storage of augmented data, as transformations are generated and applied on the fly during each training epoch. Throughout the training loop, the model weights that achieve the highest validation accuracy are saved. These weights are subsequently used to visualize the latent space, ensuring that the network has learned meaningful representations.

**Figure 3:**
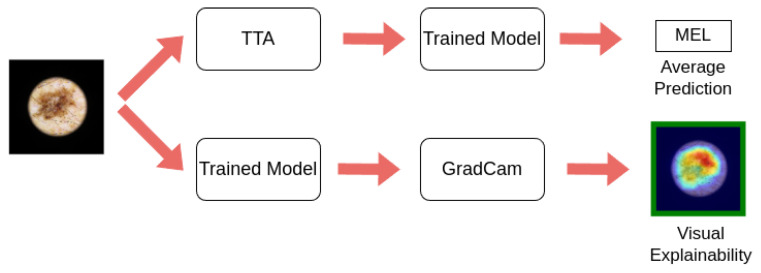
During inference, the proposed methodology generates predictions by averaging the outputs obtained through Test Time Augmentation. Additionally, it provides a visual explanation of the predictions providing a saliency map. The regions highlighted in red represent the most significant areas of the image, with importance gradually decreasing toward the blue regions, which indicate the least significant areas. This color-coding scheme is consistent across all images presented in this paper.

In Figure 3, it can be seen that, during the inference phase, Grad-CAM is utilized to generate visual explanations for each model, and Test Time Augmentation is applied to achieve more accurate predictions. Grad-CAM, a visualization technique used to interpret the predictions of CNNs, is typically applied to a single forward pass of the model. Grad-CAM is applied to the model without TTA.

### 2.3. Test Time Augmentation

**Figure 4:**
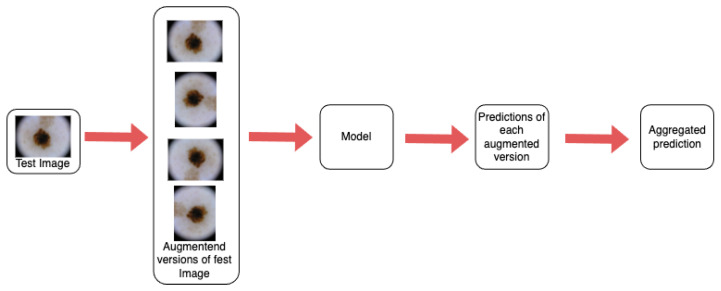
Description of the Test Time Augmentation methodology. In the images, rotation is only used for visualization purposes. This is a more detailed version of the TTA branch of Figure 3.

We use a Test Time Augmentation technique which consists of feeding transformed copies of a given image into a pre-trained model and aggregating the resulting predictions, as described in Figure 4. We assume the following:

- A pre-trained model $f:X\to R^{C}$ that maps images to vectors of class probabilities. X denotes the space of images on which the model can operate and C denotes the number of classes. This procedure is model-agnostic.
- A set of N augmentation functions, $A={\left\{a_{n}\right\}}_{n=1}^{N}$, where $a_{n}:X\to X$ is a transformation function designed to preserve class-dependent features while modifying class-independent variables, such as image scale or color balance.
- A set of M images $X={\left\{x_{m}\right\}}_{m=1}^{M}$ and the respective labels ${\left\{y_{m}\right\}}_{m=1}^{M}$ where $y_{m}\in \left\{1,\cdots ,C\right\}$. This is could be a part of the overall test set.
- *K*, a fixed number of augmentations that we decide to employ.

Based on these assumptions, we estimate the following aggregation function: $g:R^{C\times K}\to \left\{1,\cdots ,C\right\}$ which takes the vectors of predictions for all K transformed versions of the given image as input and uses them to produce an overall prediction. In this work, we assume that the N augmentations have the same weight *g* (i.e., average), and K is equal to N. We will refer to this setting of *g* as standard Test Time Augmentation. The pseudocode for the TTA procedure is delineated in Algorithm 1.
**Algorithm** **1** Test Time Augmentation employing Convolutional Neural Networks: In this study, the suite of transformations applied comprises both horizontal and vertical flips and rotations of 90, 180, and 270 degrees.1:**procedure** TTA_CNN (image, model, transformations)2:    predictions←emptylist3:    original_pred←model.predict(image)4:    predictions.append(original_pred)5:    **for** t∈transformations **do**6:        aug_image←t(image)7:        pred←model.predict(aug_image)8:        predictions.append(pred)9:    final_pred←average(predictions) **return** final_pred

### 2.4. Visual Explaining of Proposed Approach

Our aim is to employ xAI methods to highlight the skin lesion regions that mainly influence disease classification and plot the skin lesion image as a point in Cartesian fashion. In Section 2.4.1, we describe the GRAD-CAM methodology used to create a heatmap for the image region containing the skin lesions. in Section 2.4.2, the image latent space is defined, and in Section 2.4.3, we describe how this image latent space has been plotted.

#### 2.4.1. CNN Backbone Visual Explanation

For this task, we use Grad-CAM methodology [24] to generate class activation maps (CAMs) for certain outputs of a model. As described in Figure 1, the backbone consists of some convolution layers, followed by a flattening layer and then an FC (fully connected) layer. We analyze the outputs of the last convolutional layer, which is flattened and passed to the FC layer.

The output of the last convolution layer consists of K feature maps, each of width W and height H. We represent them collectively as a tensor $A\in R^{K\times W\times H}$ where $A_{k}\in R^{W\times H},1\leq k\leq K$ with *k* would be the k-th feature map. The output before passing it to softmax is an array $Y\in R^{C}$ where C=8 is the number of skin lesion classes. Choosing outputs of the last convolution layer allows us to capture high-level features. Obviously, we can use other layers of the backbone, but the initial convolutional layers capture local features and their gradients do not give any explanation about high-level features.

We apply the gradient to decode the model decision. To compute a gradient, we need a function and the variable with respect to which to calculate it. We want to find the relationship between the feature maps, $A\in R^{K\times W\times H}$, and the outputs of the backbone, $Y\in R^{C}$. Each feature map captures some high-level features of the input image and contributes to making the final decision $Y\in R^{C}$. To decode the decision of predicting the class $c\in \left\{1,\cdots ,C\right\}$, we focus on the $y_{c}$ output. We suppose that any change in the feature maps in $A\in R^{K\times W\times H}$ makes a change in the value $y_{c}$, so we compute the gradient of $y_{c}$ with respect to the k-th feature maps $A_{k}\in R^{W\times H}$. Our goal is to estimate the change in $y_{c}$ with respect to $A\in R^{K\times W\times H}$, a tensor which consists of all K feature maps. Some feature maps in A might have a greater influence in the final output $y_{c}$ than others. We want to assign a score to each of these feature maps, depending upon their influence in $y_{c}$. For this reason, we compute the average of all the elements of the gradient and use it as a score for this feature map. In other words, we compute the global average pooling (GAP) of the feature map:

(6) $$\alpha_{k}^{c}=\frac{1}{Z}\sum_{i}\sum_{j}\frac{\partial y_{c}}{\partial A_{k}^{i.j}}$$

where 1≤i≤W, 1≤j≤H and Z=W×H.

The greater the score for a feature map, the more influence it has on $y_{c}$; by increasing the value of the pixel (element) $A^{i,j}$, the value of $y_{c}$ consequently increases. In order to compose the Grad-CAM heatmap, we compute the weighted sum of all the K feature maps in A:

(7) $$S=\sum_{k=1}^{K}\alpha_{k}^{c}A_{k}$$

where $S\in R^{W\times H}$ and $\alpha_{k}^{c}$ is computed by Equation (6). Finally, we apply an element-wise ReLU operation to obtain the final heatmap:

(8) $$L_{GradCAM}^{C}=ReLU\left(S\right)$$

We apply a ReLU operation, as expressed in Equation (8), to a linear combination of maps because we are interested only in features that have a positive influence on the class of interest, i.e., pixels whose intensity should be increased in order to increase $y_{c}$.

#### 2.4.2. From Image Space to Latent Space

Finding the optimal latent space representations to classify input image samples according to their corresponding class labels is the goal of the backbone block (see Figure 1); this problem is also known as the supervised classification problem. To make things clearer, let us look at $X={\left\{x_{m}\right\}}_{m=1}^{M}$ and the labels that go with it. The set of M i.i.d data samples (images) and the labels that go with them are $y={\left\{y_{m}\right\}}_{m=1}^{M}$, as well as $f_{class}\left(X;\theta ,W,b\right)$, the classifier function, which associates the backbone model parameters’ representation θ with the dataset X and its corresponding class labels y. This classifier function is composed of two functions:

1. Backbone function $f_{bbone}\left(X;\theta \right)$, which associates the corresponding latent space representation z with dataset items X;
2. The fully connected layer function $f_{fconn}\left(z;W,b\right)$ uses the corresponding hyperplanes, denoted by W and b, to transform the set of latent space representation $z={\left\{z_{m}\right\}}_{m=1}^{M}$ to the corresponding labels in y.

This way, we have

(9) $$f_{class}\left(X;\theta ,W,b\right)=f_{fconn}\left(f_{bbone}\left(X;\theta \right);W,b\right)$$

The probability of classifying $x_{i}$ according to class labels $y_{i}$ for each data sample in X is denoted by $p(y_{i}\in y|x_{i}\in X$). The objective function for overall classifier likelihood is thus defined as follows:

(10) $$\phi \left(\theta ,W,b\right)=\underset{x,y∼p_{x,y}}{E}\left[\log p\left(y^{\left(i\right)}|x^{\left(i)\right)};W,b\right)\right]$$

Equation (10) is used to train the classifier, yielding the best estimate of the model parameters $\theta^{*}$ in terms of convergence. This gives rise to the backbone latent space representation $z_{m}^{*}$, which is defined as follows:

(11) $$z_{m}^{*}=f_{bbone}\left(x_{m};\theta =\theta^{*}\right)$$

We shall analyze these representations in latent space. Compared to the pixel space, we anticipate a more straightforward image representation from this latent space. Hopefully, the 2D Cartesian axis representation utilizing t-SNE will yield some insightful results.

#### 2.4.3. Latent Space Visualization

The first thing we desire while working with datasets is to visualize data in a meaningful way. The skin lesion image space has H×W×3 dimensions and of course cannot be plotted in an understandable way. The challenge is to squeeze all this dimensionality into something that can be grasped instantly in 2D-3D plots. t-SNE [71] helps us in this aim; it maps high-dimensional data into a space of two or three dimensions, keeping the same distance between the data points. Then, a scatter plot will allow us to see these points. In particular, it describes every high-dimensional object using a two- or three-dimensional point in a way that assigns nearby points to comparable things and distant ones to different objects.

There are two primary phases in the t-SNE algorithm.

- First, in order to provide comparable objects a high chance of being chosen and different points a very low chance of being chosen, t-SNE first creates a probability distribution over pairs of high-dimensional objects.
- Second, with regard to the locations of the points in the map, t-SNE minimizes the Kullback–Leibler divergence between the two distributions and defines a similar probability distribution across the points in the low-dimensional map.

Given a set of *N* high-dimensional latent space vectors $z_{1},\cdots ,z_{N}$, t-SNE first computes probabilities $p_{ij}$ that are proportional to the similarity of objects $z_{i}$ and $z_{j}$, as follows:

(12) $$p_{j|i}=\frac{\exp(-{∥z_{i}-z_{j}∥}^{2}/2\sigma_{i}^{2})}{\sum_{k\neq i}\exp\left(-{∥z_{i}-z_{k}∥}^{2}/2\sigma_{i}^{2}\right)}$$

As Van der Maaten and Hinton explained [71], “The similarity of datapoint $z_{j}$ to datapoint $z_{i}$ is the conditional probability, $p_{j|i}$, that $z_{i}$ would pick $z_{j}$ as its neighbor if neighbors were picked in proportion to their probability density under a Gaussian centered at $z_{i}$”.

(13) $$p_{ij}=\frac{p_{j|i}+p_{i|j}}{2N}$$

Using the [bisection approach], the bandwidth of the [Gaussian kernels] $\sigma_{i}$ is adjusted so that the [perplexity] of the conditional distribution equals a predetermined perplexity. Smaller values of $\sigma_{i}$ are utilized in denser regions of the data space, allowing the bandwidth to be adjusted to the [density] of the data.

With $y_{i}\in R^{d}$, t-SNE seeks to learn a *d*-dimensional map $y_{1},\cdots ,y_{N}$ that captures the similarities $p_{ij}$ as closely as possible. In order to achieve this, it uses a very similar method to estimate similarities $q_{ij}$ between two points in the map $y_{i}$ and $y_{j}$. Specifically, $q_{ij}$ is defined as

(14) $$q_{ij}=\frac{{\left(1+{∥y_{i}-y_{j}∥}^{2}\right)}^{-1}}{\sum_{k\neq m}{\left(1+{∥y_{k}-y_{m}∥}^{2}\right)}^{-1}}$$

To allow dissimilar items to be modeled far away in the map, here, we utilize a heavy-tailed [Student-t distribution] (with one degree of freedom, which is the same as a [Cauchy distribution]) to quantify similarities between low-dimensional points.

By minimizing the (non-symmetric) [Kullback–Leibler divergence] of the distribution *Q* from the distribution *P*, the positions of the points $y_{i}$ in the map are found, that is,

(15) $$KL\left(P||Q\right)=\sum_{i\neq j}p_{ij}\log\frac{p_{ij}}{q_{ij}}$$

Gradient descent is used to minimize the Kullback–Leibler (KL) divergence with regard to the points $y_{i}$. A map that accurately captures the commonalities among the high-dimensional inputs is the outcome of this optimization.

### 2.5. Dataset

The ISIC Challenge provided the dataset used in this study [26,72,73]. The 25,331 JPEG images of skin lesions comprise the following categories: actinic keratosis (AK; 866 images), basal cell carcinoma (BCC; 3323 images), benign keratosis (solar lentigo/seborrheic keratosis/lichen planus-like keratosis) (BKL; 2624 images), dermatofibroma (DF: 239 images), melanoma (MEL; 4522 images), melanocytic nevus (NV: 12,875 images), squamous cell carcinoma (SCC; 628 images), and vascular lesions (VASC; 253 images). With an uneven quantity of photos in each class, this dataset is one of the hardest to divide into eight classes. Following a random shuffle, this dataset is split using the splitting partitions 80%-10%-10% for the training, validation, and test datasets.

### 2.6. Implementation Details

As described in Section 2.2, we fine-tuned very common CNNs such as ResNeXt-50 [31], Resnet-152 [32], and EfficientNet [33] B0 to B7 for the skin classification target task, starting from the large “upstream” ImageNet dataset [13]. We did not report the results of EfficientNet from B0 to B3 because they are too small for solving such a complex task.

All the networks were trained using the same set of hyperparameters, as summarized in Table 1. These hyperparameters were optimized in our previous work, where an extensive process was undertaken to select the most appropriate set of data augmentation techniques, optimization procedures, and image sizes. This meticulous approach resulted in improved performance compared to other studies [74]. Among the evaluated optimization methods, the Momentumized, Adaptive, Dual-Averaged Gradient Method for Stochastic Optimization (MADGRAD) [75] achieved the best performance. Notably, MADGRAD has been successfully applied in CNN-based architectures, as illustrated in [76]. This optimizer outperforms both Stochastic Gradient Descent (SGD) and ADAM, even in tasks where other adaptive methods typically underperform. As a member of the AdaGrad family, MADGRAD introduces several modifications that enhance its effectiveness in deep learning optimization problems. The batch size, which indicates the number of images used before each update of the CNN weights, represents a critical hyperparameter. However, despite its importance, we did not tune the batch size in this study. For the parameters of the learning rate scheduler, the patience is the number of epochs to wait without improvement of the validation loss before multiplying the current learning rate by the factor. This is carried out until the minimum learning rate is reached. For the parameters of the optimizer, the weight decay is a regularization term that prevents the network from overfitting by encouraging smaller weights. In particular, weight decay (wd) refers to an $L_{2}$ regularization term that is added to the loss function. This term penalizes large weights by adding the squared magnitude of all weights to the loss. The inclusion of weight decay helps prevent overfitting by discouraging the model from becoming excessively complex, thereby promoting generalization to unseen data. Momentum makes the optimizer also take into account the past optimization step. The maximum number of training epochs was set to 200 for each CNN backbone; however, none of the models required training for the full duration. We employed early stopping, a technique that halts training when the validation metric ceases to improve. This approach mitigates the risk of overfitting and ensures efficient use of computational resources.

Table 2 details other parameters that change for all the evaluated networks. This table reports input image size, the output of the last layer’s tensor dimensions, and the relative input of the fully connected layer, which vary accordingly with the different CNN backbones we investigated. The last backbone layer includes Adaptive Average Pooling, which reduces the input tensor from (w×h×c) dimensions to a tensor of fixed dimensions (1×1×c), which establishes the input dimension of the fully connected layer.

### 2.7. Data Augmentation

An identical set of data augmentations was used to train each network. The majority of the time, we employed affine transformation, random rotation, and random flipping of the horizontal and vertical planes.

Both Table 3 and Table 4 include the parameters that were utilized to create the collection of images that were augmented to train the networks. The parameters for augmentation are derived from our previous work [74]. The affine transformation had the following parameters: shear from −30 to 30 degrees, and scaling factor from 0.7 to 1.7. Instead of sampling from a distribution of 0.2 variance for brightness and contrast, hue and saturation had a variance of 0.05. The network performance deteriorates due to random grayscale augmentation, which is rather common. This is probably due to the fact that the color is important for disease classification; indeed, it is one of the major differences between nevus and melanoma. Gaussian noise and Gaussian blur were also tried but none of these augmentations improved the overall performance. Due to the stochastic nature of these augmentations, each image in the original dataset can generate numerous unique augmented versions. Throughout the training process, with augmentations applied randomly to each image in every epoch, the appearance of an image may theoretically differ in each epoch. This comprehensive augmentation strategy substantially increases the diversity of the training data.

### 2.8. Metrics

To evaluate the performance of the classifiers, we used the most common quantitative metrics, considering (i) true positive (TP): correct prediction of positive class; (ii) true negative (TN): correct prediction of negative class; (iii) false positive (FP): incorrect prediction of positive class; and (iv) false negative (FN): incorrect prediction of negative class. Accuracy (Acc.), precision, recall and $F_{1}$ score are calculated as below:

(16) $$Accuracy=\frac{TP+TN}{FP+TN+TP+FN}$$

(17) $$Precision=\frac{TP}{TP+FP}$$

(18) $$Recall=\frac{TP}{TP+FN}$$

The precision and recall harmonic means are combined to obtain the F1 score, which is a predictive performance measure; it symmetrically expresses recall and precision in a single metric (see Equation (19)).

(19) $$F_{1}=\frac{2}{\frac{1}{\;\mathrm{Recall}\;}+\frac{1}{\;\mathrm{Precision}\;}}=2\frac{\;\mathrm{Precision}\;\cdot \;\mathrm{Recall}\;}{\;\mathrm{Precision}\;+\;\mathrm{Recall}\;}=\frac{2\mathrm{TP}}{2\mathrm{TP}+\mathrm{FP}+\mathrm{FN}}$$

### 2.9. Hardware and Software

This research study was conducted using the Python 3.7 programming language with pytorch 2.3 libraries. The models were trained on a workstation equipped with four NVIDIA RTX TITAN GPUs (24 GB each) with 64 GB RAM and an Intel i7 Processor.

## 3. Results

In this section, the results of the performed experiments are presented.

### Ablation Study

This section addresses the subject of how TTA affects the accuracy performance of the different network architectures. We assess the performance of the various investigated network architectures, both with and without the TTA algorithm, through ablation investigations.

Table 5 summarizes the results obtained using the proposed approach introduced in Section 2. We fine-tuned six different architectures following the strategy described in Section 2.2. The ablation study, as indicated in Table 5, demonstrates that the accuracy increased by around 0.3% when the TTA method was used with the EfficientNet-B6 CNN backbone. As can be observed, every backbone that has been studied produces extremely positive results (ranging from 97.04%) in terms of accuracy.

As we proceed with the development of this ablation investigation, we discover changes, as indicated in the “Prediction Changed“ column of Table 5, which lists the quantity of skin disease images that underwent image processing and had their categorization label altered following the TTA application. The “Corrections” column shows the number of previously incorrectly classified images that have been allocated to the right class following the use of the TTA.

Correspondingly, the number of previously correctly classified images that are misclassified after applying the TTA is listed in the “Introduced Error” column. Observations reveal that, on average, there are approximately 100 label changes, which aligns with the findings presented in [77]. This small number of label changes is due to both the low variability of the image content and the uniqueness of the context in which they are acquired. These results validate that the TTA plays a role in increasing the performance of skin disease categorization in all analyzed topologies. The difference between the “Corrections” and “Introduced error” columns (the final column, “Difference”), which is always non-negative, is evidence of this. The reason for this outcome is that there was not an unreasonably high amount of training images, as supported by the findings in [77]. We can confirm that applying TTA to the studied backbones enhances performance at a rate ranging from 0.15% to 0.33%, with the exception of one case (ResNet152) where no improvement in performance is observed. TTA has therefore had a generally beneficial effect. The improvement arises because Test Time Augmentation (TTA) leverages diverse augmented views of the same test image, enabling the model to aggregate predictions across these variations.

Detailed performance declines for each skin disease category for the architecture that gives the best performance are reported in Table 6 and Table 7. As can be seen in Table 6, the mean precision of the our best architecture without TTA is around 0.84, the balance multi-class accuracy is 97.31%, and the mean F1 score is 0.85. Using TTA with the same model average precision increases the average F1 score to 0.86 and the BCA to 97.58%, as reported in the last row of Table 7. These results confirm the positive contribute of the TTA method, and they are encouraging considering the complexity of the skin disease classification task.

**Figure 5:**
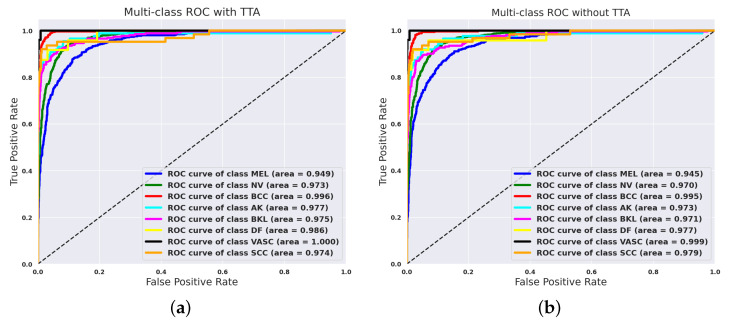
ROC curves for all classes using EfficientNet-B6 without (**left**) and with (**right**) Test Time Augmentation. (**a**) Without Test Time Augmentation. (**b**) With Test Time Augmentation.

In Figure 5, the impact of using Test Time Augmentation on the ROC-AUC metric is illustrated. While the average metric remains largely unchanged, likely due to the low error rate, an improvement in the ROC-AUC score is observed for the most critical classes, such as melanoma (MEL) and basal cell carcinoma (BCC) which represent malignant cancers. This enhancement could be significant in real-world applications.

**Figure 6:**
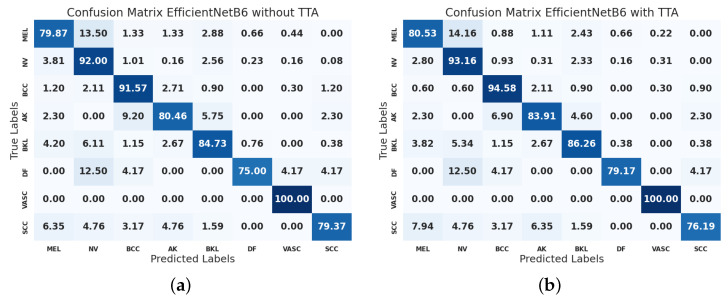
Confusion matrices of EfficientNet-B6 without (**left**) and with (**right**) Test Time Augmentation. The values are normalized per row. (**a**) Without Test Time Augmentation. (**b**) With Test Time Augmentation.

Figure 6 depicts two confusion matrices for EfficientNet-B5, illustrating its performance on a classification task without and with TTA. The confusion matrices are normalized per row, allowing for easier comparison of class-wise performance. The left confusion matrix shows a strong diagonal presence, indicating that the model has a high accuracy in correctly classifying the majority of the classes. Some classes, such as DF and SCC, show relatively higher misclassification rates. The application of TTA has led to noticeable improvements in classification accuracy for several classes that enhance diagonal dominance, suggesting that TTA contributes to better model confidence and accuracy. Classes that initially had higher misclassification rates, such as DF and SCC, exhibit reduced rates, indicating that TTA helps the model better distinguish between these classes. The use of Test Time Augmentation in EfficientNet-B5 enhances the model’s classification performance, leading to higher accuracy and reduced misclassification rates across various classes. This indicates that TTA is a valuable technique for improving the robustness and reliability of deep learning models in this image classification tasks.

We have also estimated the execution times for each model using a CPU, along with their corresponding standard deviations. Table 8 demonstrates that the implementation of TTA leads to an increase in inference times; however, this increase is marginal, remaining within the range of a few seconds. This additional time is negligible when compared to the overall duration required for a comprehensive dermatological examination.

## 4. Discussion

Table 9 presents the summary of the works described in Section 1.1. It can be observed that most of the researchers have not used the ISIC 2019 dataset due to its large number of images. We also observe that the most commonly employed explainability method is CAM [25], but it typically exhibits moderate accuracy. Furthermore, in this table, we demonstrate that our model achieves better or comparable BCA performance on the eight-class ISIC 2019 dataset, reaching 97.58%. We also discuss our results using Grad-CAM as an xAI method as described in Section 4.1 and visualize the latent space using t-SNE as detailed in Section 4.2.

### 4.1. How Does CNN Classify Skin Diseases? Using xAI for Exploring the CNN Layers

In risk-sensitive fields such as medical imaging, where a false negative prediction can make a difference between life and death, it is crucial to evaluate the model’s trustworthiness. GradCAM [24], as described in Section 2.4.1, provides practitioners and researchers with an intuitive heatmap of the important image regions. Heatmap explanations require human interpretation. Although a region is highlighted or similar images are displayed, the clinical evaluator must still determine why the region is relevant or why the images are similar. This introduces confirmation bias, leading humans to hypothesize that a classifier uses the same features they would, when presented with a plausible xAI explanation—although they cannot know this with certainty.

Fine-grained heatmaps can slightly alleviate this issue by showing what structure(s) the model’s decision is based on as precisely as the image resolution permits. It is important to consider that even the fine-grained heatmaps are not a “silver bullet”. They only help with data interpretation, but do not change the fact that clinician evaluation remains necessary.

**Figure 7:**
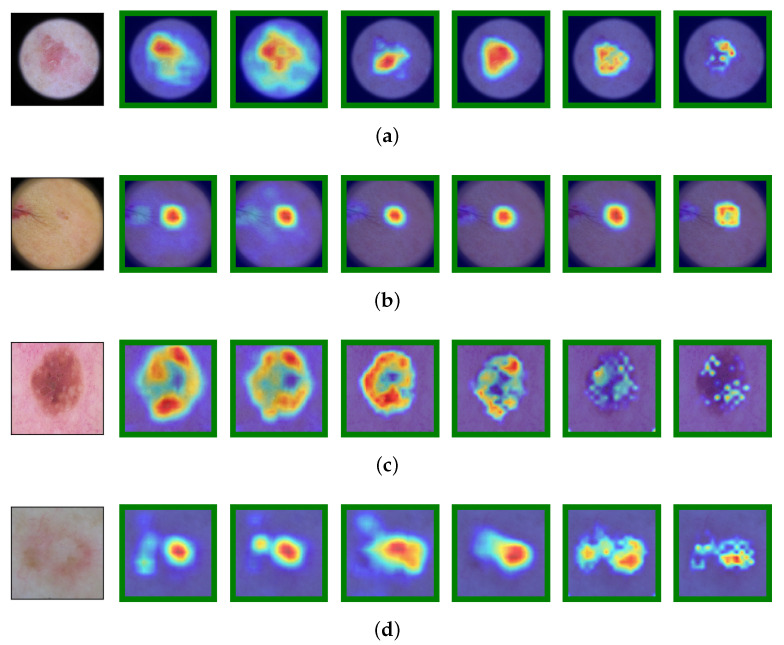
Images and corresponding Grad-CAM outputs for the best-performing networks. From left to right: ResNeXt-50, ResNet-152, EfficientNet-B4, EfficientNet-B5, EfficientNet-B6, and EfficientNet-B7. Green border indicates the correct classification. (**a**) An image of actinic keratosis (AK). All neural networks correctly classify the image, despite focusing on different parts of the image. (**b**) An image of basal cell carcinoma (BCC). All predictions are made by considering the diagnostically significant regions of the image. However, some networks, such as ResNeXt-50 and ResNet-152, are also influenced by less relevant pixels. (**c**) An image of benign keratosis (BKL). All neural networks correctly classify the image, although EfficientNet-B6 and EfficientNet-B7 partially disregard certain areas of the lesion. (**d**) An image of dermatofibroma (DF). The regions of the image that determine the classification vary between different models.

**Figure 8:**
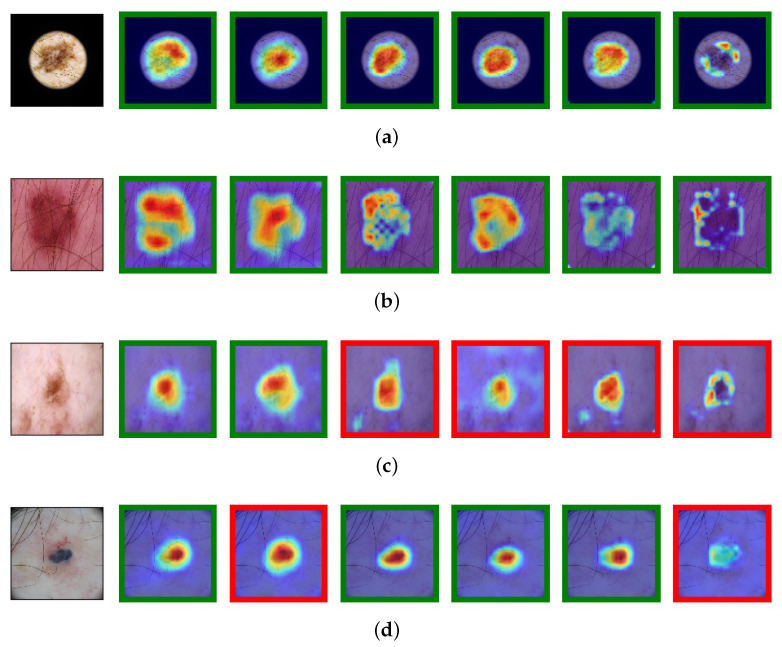
Images and corresponding Grad-CAM outputs for the best-performing networks. From left to right: ResNeXt-50, ResNet-152, EfficientNet-B4, EfficientNet-B5, EfficientNet-B6, and EfficientNet-B7. The red border indicates misclassification; the green border indicates correct classification. (**a**) An image of melanoma (MEL). The EfficientNet-B7 correctly classifies the image despite focusing on only a few pixels of the lesion. (**b**) An image of melanocytic nevus (NV). All neural networks correctly identify the lesion, but each network bases its decision on different regions of the image. (**c**) An image of squamous cell carcinoma (SCC). All EfficientNet models misclassify this image, ResNet CNNs are able to correctly classify the image. (**d**) An image of a vascular lesion (VASC). Although all models base their predictions on the pixels corresponding to the lesion, the larger models, ResNet-152 and EfficientNet-B7, misclassify the image.

Bearing in mind the above statements, Figure 7 and Figure 8 show that most of the time, models highlight the part of the image that positively influences the correct classification of the skin image. As previously mentioned, physicians can help researchers develop more accurate classifiers by interpreting these attention heatmaps.

**Figure 9:**
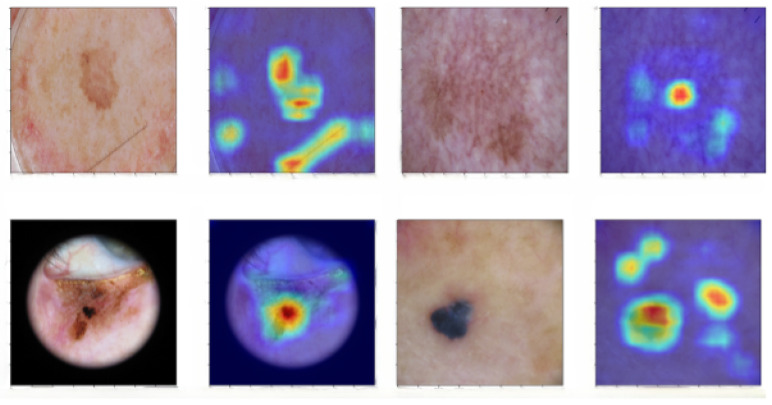
Examples of wrongly classified images together with their corresponding Grad-CAM heatmaps. In order, from top-left to bottom-right: two nevus cases classified as BKL, BKL classified as melanoma, and BCC classified as melanoma.

Another interesting neural network study can be performed using Grad-CAM on misclassified images. As can be seen in Figure 9, the network usually focuses on the lesion itself in misclassified images; however, it can be distracted by external elements, as illustrated in the first example. In this image, the network pays attention to the straight line made by the doctor. In the second image, the network looks at only one part of the skin issue, which could lead the network to make a wrong classification by focusing on only a few elements of the skin lesion. In the last image, the network focused on a healthy skin region, but it is just a part of the skin of the patient. In the third image, the network is looking at the right part of the image but, nevertheless, is not able to correctly classify the image.

### 4.2. Image Plotting Using Latent Space Representation

As described in Section 2.4.3, in order to explore the visual characteristics of the eight different skin disease classes, we examine internal features learned by the proposed model.

**Figure 10:**
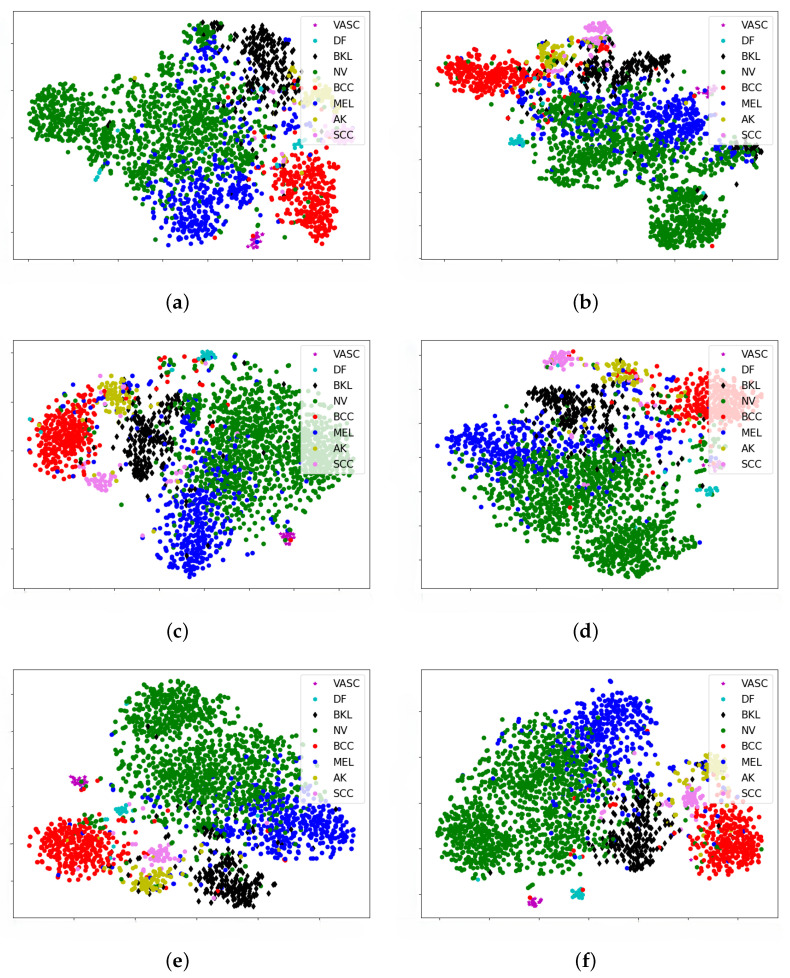
Latent spaces of the best-performing networks for each backbone architecture. Images belonging to the same class are positioned closely in the embedding space. Clusters corresponding to diseases with similar visual features are situated nearer to each other compared to clusters of visually distinct diseases. This behavior is consistent across all backbone architectures. (**a**) Latent space of ResNeXt50. (**b**) Latent space of ResNet152. (**c**) Latent space of EfficientNetB4. (**d**) Latent space of EfficientNetB5. (**e**) Latent space of EfficientNetB6. (**f**) Latent space of EfficientNetB7.

As demonstrated in Figure 10, each point represents a skin image projected from the N-dimensional output of the last hidden layer of the proposed architecture into two dimensions (N varying according to the chosen backbone; Table 2). Clusters of points from the same clinical class are clearly visible.

Classes that share similarities, such as nevus (NV) and melanoma (MEL), are depicted as closer together in all the subfigures of Figure 10. Furthermore, these categories are split into two or more clusters representing the real scenario where many different subtypes of nevus and melanomas can be found. For example, we speculate that one of the melanoma clusters consists of amelanotic melanomas, a subtype that does not produce melanin, which gives most melanomas their dark appearance. This means that they do not look like other melanomas. Instead, they may appear skin-colored, pink, or even reddish, with gray or brownish edges. Interestingly, this information was not present in the training dataset; the network learned this autonomously. Unfortunately, it is difficult to find high-quality, labeled data of different subcategories of skin problems, so we could not conduct a statistically relevant study. By examining all the plots, we observe that classes that are close in one latent space are close in others: basal cell carcinomas (red dots) are always close to actinic keratoses (AK) (yellow dots).

**Figure 11:**
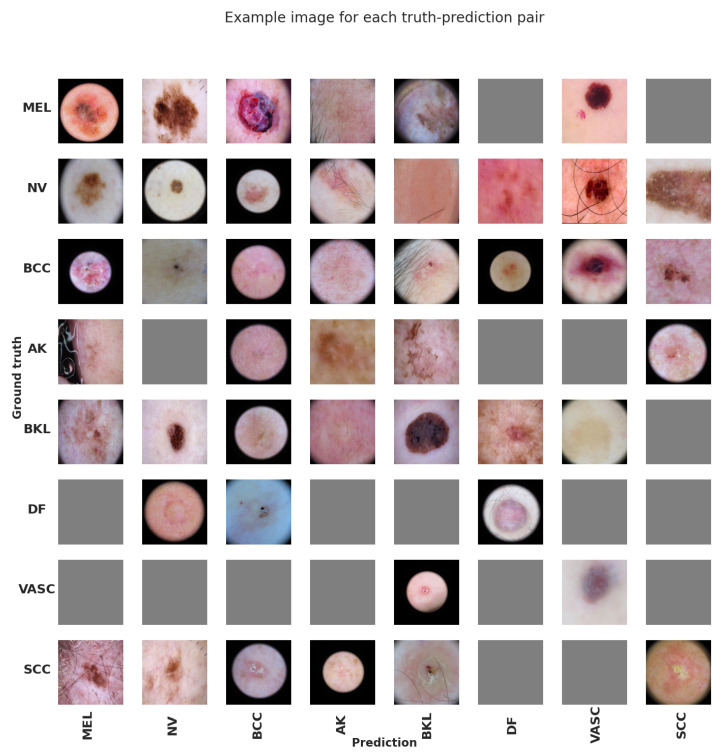
Qualitative analysis of the model’s performance. The images are organized such that rows represent the ground truth labels, while columns correspond to the predictions made by the EfficientNet-B6 model. In instances where there are no images for a particular ground truth–prediction pair, a grey placeholder image is displayed.

Figure 11 presents a qualitative evaluation of the best-performing model. While a more detailed analysis in collaboration with a group of dermatologists is warranted, it is evident that many incorrect predictions arise from the low quality of the input images. For instance, actinic keratosis (AK) misclassified as melanoma (MEL) can be attributed to the presence of a gel obscuring the skin lesion. Similarly, a nevus misclassified as squamous cell carcinoma (SCC) appears to result from the incomplete capture of the lesion within the image.

## 5. Conclusions

In this study, we have addressed the challenge of automated skin disease classification using CNNs and TTA. To ensure the reliability of our proposed approach, we utilized a set of xAI tools. Our methodology achieved a Balanced Classification Accuracy (BCA) of 97.58%, which is either superior or comparable to the most cited works in the literature on the eight-class ISIC 2019 dataset. Furthermore, our experiments confirmed that the TTA approach enhances classification accuracy. To evaluate the proposed architectures, we employed Grad-CAM and t-SNE. Grad-CAM was used to generate visual explanations, highlighting the important regions in skin lesion images that the network focused on when making predictions. This interpretability tool provides insights into the model’s decision-making process, making it more transparent and understandable for clinicians. Additionally, t-SNE was applied to project the high-dimensional latent space of the CNN into a two-dimensional Cartesian plane, facilitating the visualization of the clustering patterns of different skin lesions in the hidden space. This visualization aids physicians and dermatologists by offering an intuitive representation of how the network internally organizes and differentiates skin lesion features, thereby supporting diagnostic decision-making. Future work will focus on exploring the reasons behind the consistent misclassification of certain skin disease images across all investigated architectures. We also aim to extend our experiments by incorporating additional data attributes, such as the patient’s gender, age, race, and the anatomical location of the lesion (e.g., neck, upper body, lower body). Moreover, we plan to conduct further experiments to enhance network performance, including experimenting with alternative augmentation strategies during TTA. Furthermore, instead of relying on conventional backbones, the utilization of Convolutional Kolmogorov–Arnold Networks (Convolutional KANs) can offer a superior backbone for skin classification tasks, as they have demonstrated enhanced performance with fewer parameters on well-known datasets [81]. Specifically, the U-Net architecture [82] has been modified by integrating dedicated Kolmogorov–Arnold Network (KAN) layers [83], resulting in a more accurate model with reduced computational cost [84,85]. Despite these promising results, particularly in the field of medical imaging, such techniques have not yet been applied to skin classification and segmentation. The potential of Convolutional KANs lies in their theoretical foundation based on the Kolmogorov–Arnold representation theorem. By reducing the number of parameters while maintaining high performance, these networks can alleviate computational burdens and facilitate deployment in resource-constrained environments. Another avenue for improvement could involve developing an algorithm based on Physics-Informed Neural Networks (PINNs), which are neural networks trained to solve supervised learning tasks while respecting given laws of physics described by general nonlinear partial differential equations (PDEs) [86], such as SquareResNet [87]. PINNs have been widely used for medical tasks [88] due to their ability to incorporate physical knowledge into the learning process, potentially leading to models that are more generalizable, robust, and interpretable. These approaches could lead to more efficient, accurate, and generalizable models by leveraging theoretical advancements and incorporating domain-specific knowledge into the learning process. A primary limitation of our study is the absence of an evaluation of the proposed method on out-of-distribution images. Previous research has underscored the importance of this concern in skin disease classification, as variations in imaging equipment and the expertise of the physician capturing the images may impact the image features. Thus, a comprehensive evaluation using out-of-distribution images is crucial to accurately assess the model’s performance in real-world clinical settings, especially when considering the diversity of Fitzpatrick skin types. Another limitation of this study is the absence of cross-validation in the evaluation process, which would provide a more robust assessment of the model’s generalizability. We plan to address this limitation in future work.

## Figures and Tables

**Table 1:** This table outlines the key hyperparameters used uniformly across all backbone models during training. The configuration details are organized into three main sections: training, scheduler, and optimizer, providing a concise but comprehensive summary of the hyperparameter setup.

Training		Scheduler ReduceLRonPlateau			Optimizer MADGRAD		
**Batch Size**	**Loss**	**Factor**	**Patience**	**Min LR**	**Learning Rate**	**Weight Decay**	**Momentum**
512	WCE	0.1	10	$1\times 10^{-7}$	0.001	0	0.9

**Table 2:** The size of the input image and the dimensions of the input to the final convolutional layer are specified. Following this convolutional layer, a fully connected layer is appended to produce outputs corresponding to the eight skin disease classes, with each class representing a distinct condition.

Backbone	Image Size	w × h × c	Input Fully Connected
ResNeXt50	600 × 600 × 3	19 × 19 × 2048	2048
ResNet152	600 × 600 × 3	19 × 19 × 2048	2048
EfficientNet-B4	380 × 380 × 3	12 × 12 × 1792	1792
EfficientNet-B5	456 × 456 × 3	15 × 15 × 2048	2048
EfficientNet-B6	528 ×528 × 3	17 × 17 × 2304	2304
EfficientNet-B7	600 × 600 × 3	19 × 19 × 2560	2560

**Table 3:** Parameters for geometric transformations used during data augmentation. This table outlines the probabilities and ranges for horizontal and vertical flips, scaling, rotation, and shearing applied to the input data to enhance model generalization and robustness.

Random H. Flip	Random V. Flip	Random Scale		Random Rotation		Random Shear	
**Probability**	**Probability**	**From**	**To**	**From**	**To**	**From**	**To**
0.5	0.5	0.7	1.7	0 degrees	359 degrees	−30	30

**Table 4:** Parameters for color adjustments applied as part of data augmentation. These settings specify the maximum perturbations in brightness, contrast, hue, and saturation, enhancing model robustness to varying lighting and color conditions.

Brightness	Contrast	Hue	Saturation
0.2	0.2	0.05	0.05

**Table 5:** Performance metrics across different model backbones with and without TTA. The table compares baseline accuracy with TTA-enhanced accuracy and highlights the number of predictions changed, corrections made, and errors introduced due to TTA. EfficientNet-B6 achieved the highest accuracy with TTA. The row corresponding to the model achieving the best performance is highlighted in bold for emphasis.

Backbone	Accuracy	TTA	Predictions	Corrections	Errors	Difference
		**Accuracy**	**Changed**		**Introduced**	
ResNeXt50	97.10%	97.39%	82	52	22	30
Resnet 152	96.94%	96.94%	93	40	40	0
EfficientNet-B4	97.12%	97.27%	105	55	39	16
EfficientNet-B5	97.06%	97.39%	106	65	31	34
**EfficientNet-B6**	**97.31%**	**97.58%**	**102**	**59**	**32**	**27**
EfficientNet-B7	97.04%	97.30%	113	67	40	27

**Table 6:** Performance metrics for skin disease classification using the best model (EfficientNet-B6) without TTA. The table provides =Precision, Recall, F1 score, and accuracy for each disease class, showing strong overall performance. Mean values summarize the model’s effectiveness in predicting diverse skin disease categories under standard conditions without TTA enhancements.

Skin Disease	Precision	Recall	F1-Score	Accuracy
MEL	0.86	0.78	0.82	93.8%
NV	0.92	0.94	0.93	93.00%
BCC	0.92	0.92	0.92	97.98%
AK	0.76	0.79	0.78	98.42%
BKL	0.83	0.87	0.85	96.8%
DF	0.73	0.79	0.76	99.52%
VASC	0.77	0.96	0.86	99.68%
SCC	0.91	0.79	0.85	99.28%
Mean	0.84	0.85	0.85	97.31%

**Table 7:** Performance metrics for skin disease classification using the best model (EfficientNet-B6) with TTA. The table reports precision, recall, F1 score, and accuracy for each disease class, highlighting balanced performance across classes. The mean values demonstrate the model’s overall effectiveness in handling a diverse set of skin disease categories.

Skin Diseases	Precision	Recall	F1-Score	Accuracy
MEL	0.89	0.79	0.84	94.59%
NV	0.93	0.95	0.94	93.68%
BCC	0.93	0.93	0.93	98.14%
AK	0.77	0.84	0.80	98.58%
BKL	0.84	0.87	0.86	97.00%
DF	0.83	0.79	0.81	99.64%
VASC	0.79	92	0.85	99.68%
SCC	0.91	0.81	0.86	99.33%
Mean	0.86	0.86	0.86	97.58%

**Table 8:** The inference time for each model, with and without TTA, is evaluated using a CPU. Notably, although TTA involves 16 different versions of each input image, the execution time does not scale linearly by a factor of 16. This efficiency is achieved through an optimized method of feeding the images into the model. However, achieving a similar level of efficiency is more challenging when employing ensemble methods.

Model	No TTA (s)	TTA (s)
EfficientNetB4	0.66 ± 0.22	1.44 ± 0.09
EfficientNetB5	1.11 ± 0.26	6.76 ± 0.64
EfficientNetB6	1.66 ± 0.3	11.82 ± 0.87
EfficientNetB7	2.46 ± 0.33	15.64 ± 0.95
ResNext50	0.43 ± 0.17	4.68 ± 0.45
Resnet152	0.66 ± 0.22	4.02 ± 0.25

**Table 9:** Performance comparison with current state-of-the-art methods. The method achieving the highest test accuracy for each dataset is highlighted in bold. For the ISIC 2019 dataset, the proposed approach demonstrates either superior or comparable performance relative to other methodologies, including comparisons with more recent architectures such as the ViT.

Study	Dataset(s)	Methodology	Results	Skin Classes	xAI Method	Train:Val:Test
[35]	HAM10000	Custom CNN	Acc 82.7%	7 Classes	CAM	80%:10%:10%
[36]	ISIC 2018	CNN	ROC-AUC 94%	7 Classes	Backpropagation	Not Available
[56]	ISIC 2017	CNN	-	3 Classes	CAM	Not Available
[38]	ISIC 2018	VGG16+ResNet50	Acc 85%	7 Classes	Occlusion	70%:10%:20%
[78]	ISIC 2019	VGG16+ResNet50	Acc 72.2%, 76.7%	8 Classes	GradCAM	Not Available
[79]	1021 images	ResNet50	Acc 60.94%	4 Classes	CBIR	Not Available
[10]	**ISIC 2017, PH2**	**Modified deep CNN**	**Acc 90.4%**	**3 Classes**	**CAM**	**2000:150:600**
[57]	ISIC 2017	ResNet50	Acc 83%	2 Classes	CAM	2000:150:600
[80]	HAM10000	Inception	Acc 85%	2 Classes	GradCAM, Kernel SHAP	Not Available
[58]	ISIC 2016	VGG16	ROC-AUC 81.18%	2 Classes	CAM	900:NA:379
[12]	ISIC 2018	CNN	Spec 86.5%	1 Class	No	12378:1259:100
[11]	ISIC 2019	Deep CNN	Acc 94.92%	8 Classes	No	80%:10%:10%
[41]	**ISIC 2018**	**ResNet50**	**Acc 92%**	**7 Classes**	**No**	**70%:NA:30%**
[42]	**PH2**	**CNN**	**Acc 93%**	**3 Classes**	**No**	**70%:20%:10%**
[43]	ISIC 2018	inception V3+ResNet50	Acc 89.9%	7 Classes	No	80%:20%:NA
[44]	ISIC 2018	Deep CNN	Acc 96.67%	2 Classes	No	Variable
[45]	PH2, ISBI 2017	YOLO, Grab Cut	Acc 93.39%	3 Classes	No	2000:150:600
[46]	ISIC 2018	Custom CNN	Acc 87.5%	7 Classes	TSNE	10015:193:1512
[47]	HAM10000	Custom CNN	Acc 91.6%	7 Classes	No	8012:NA:2003
[48]	HAM10000	Custom CNN	Acc 95,73%	7 Classes	No	80%:NA:20%
[49]	**HAM10000**	**Custom CNN**	**Acc 97.96%**	**7 Classes**	**No**	**80%:NA:20%**
[50]	HAM10000	Custom CNN	Acc 96.12%	7 Classes	No	8010:NA:2005
[62]	ISIC 2019	CNN	Acc 96.12%	8 Classes	No	75%:0%:25%
[63]	ISIC 2020	Custom model	Acc 93.75%	2 Classes	No	2302:NA:989
[61]	ISIC 2019	Custom model	Acc 96.22%	8 Classes	Interpretable model	10-fold cv
[66]	ISIC 2019	Trasformers model	Acc 97.48%	8 Classes	No	80%:10%:10%
[67]	ISIC 2019	ViT	Acc 96.97%	8 Classes	No	Not Available
[67]	**ISIC 2020**	**ViT**	**Acc 97.73%**	**2 Classes**	**No**	**Not Available**
[69]	ISIC 2020	CNN + Classifier	Acc 88%	2 Classes	GradCam	70%:0%:30%
Ours	ISIC 2019	Deep CNN	Acc 97.31%	8 Classes	GradCam, TSNE	80%:10%:10%
**Ours(TTA)**	**ISIC 2019**	**Deep CNN**	**Acc 97.58%**	**8 Classes**	**No**	**80%:10%:10%**

## Data Availability

The data presented in this study are openly available in [https://challenge.isic-archive.com/data/#2019].
